# Bamboo lignocellulose degradation by gut symbiotic microbiota of the bamboo snout beetle *Cyrtotrachelus buqueti*

**DOI:** 10.1186/s13068-019-1411-1

**Published:** 2019-04-01

**Authors:** Chaobing Luo, Yuanqiu Li, Ying Chen, Chun Fu, Wencong Long, Ximeng Xiao, Hong Liao, Yaojun Yang

**Affiliations:** 1 Bamboo Diseases and Pests Control and Resources Development Key Laboratory of Sichuan Province, Leshan Normal University, No. 778, Riverside Road, Central District, Leshan, 614000 Sichuan China; 2 College of Food and Biological Engineering, Xihua University, Chengdu, 610039 Sichuan China

**Keywords:** Lignocellulose degradation, Bamboo, Gut symbiotic microbiota, *Cyrtotrachelus buqueti*, 16sRNA-sequencing

## Abstract

**Background:**

Gut symbiotic microbiota plays a critical role in nutrient supply, digestion, and absorption. The bamboo snout beetle, *Cyrtotrachelus buqueti*, a common pest of several bamboo species, exhibits high lignocellulolytic enzyme activity and contains various CAZyme genes. However, to date, no studies have evaluated the role of gut symbiotic microbiota of the snout beetle on bamboo lignocellulose degradation. Therefore, the present study investigated the role of gut symbiotic microbiota of *C*. *buqueti* on bamboo lignocellulose degradation.

**Results:**

Gut symbiotic microbiota of female (CCJ), male (XCJ), and larvae (YCJ) beetles was used to treat bamboo shoot particles (BSPs) in vitro for 6 days. Scanning electron microscopy (SEM) revealed significant destruction of the lignocellulose structure after treatment, which was consistent with the degradation efficiencies of CCJ, XCJ, and YCJ for cellulose (21.11%, 17.58% and 18.74%, respectively); hemicellulose (22.22%, 27.18% and 34.20%, respectively); and lignin (19.83%, 24.30% and 32.97%, respectively). Gut symbiotic microbiota of adult and larvae beetles was then identified using 16sRNA sequencing, which revealed that four microbes: *Lactococcus*, *Serratia*, *Dysgonomonas* and *Enterococcus*, comprise approximately 84% to 94% of the microbiota. Moreover, the genomes of 45 *Lactococcus*, 72 *Serratia*, 86 *Enterococcus* and 4 *Dysgonomonas* microbes were used to analyse resident CAZyme genes. These results indicated that gut symbiotic microbiota of adult and larvae *C*. *buqueti* is involved in the lignocellulose degradation traits shown by the host.

**Conclusions:**

This study shows that the gut symbiotic microbiota of *C*. *buqueti* participates in bamboo lignocellulose degradation, providing innovative findings for bamboo lignocellulose bioconversion. Furthermore, the results of this study will allow us to further isolate lignocellulose-degrading microbiota for use in bamboo lignocellulose bioconversion.

**Electronic supplementary material:**

The online version of this article (10.1186/s13068-019-1411-1) contains supplementary material, which is available to authorized users.

## Background

Insects are the largest group of organisms on earth, and most insect species feed on plants. Some insects, such as termites and beetles, consume plant cell walls and are involved in the degradation of lignocellulose and other types of biomass; thereby contributing to lignocellulose bioconversion and energy utilisation [1, 2]. To date, many insects, including termites, wood-feeding roaches, beetles, wood wasps, leaf-shredding aquatic insects, silverfish and leaf-cutting ants, have been shown to exhibit lignocellulose degradation ability [1].

In phytophagous insects, such as termites, beetles and wood bees, lignocellulose digestion requires cooperation between insects and symbiotic microorganisms in the insect gut, especially bacteria, protozoa, fungi, and yeasts [3]. In *Costelytra zealandica* (New Zealand grass grub), various hindgut bacteria participate in lignocellulose degradation [4] and some lignocellulose-degrading bacteria have been isolated from the larvae of the scarab beetle *Pachnoda marginata* [5]. Moreover, bacteria exhibiting cellulose hydrolysis activity have been isolated from other insects, such as *Ips pini* (North American pine engraver), *Dendroctonus frontalis* (Southern pine beetle), *Saperda vestita* (Linden borer) [6] and *Tipula abdominalis* (giant crane fly) [7]. Symbiotic gut microbes of the fungus-cultivating termites *Macrotermes annandalei*, *Odontotermes yunnanensis* and *Macrotermes barneyi* possess various lignocellulolytic enzyme genes [8]. A recent study identified 111 glycoside hydrolase (GH) family genes among the symbiotic microbes of *Macrotermes natalensis* [9]. Taken together, these studies show that there is significant involvement of insect symbiotic microbes in lignocellulose degradation.

16S rRNA sequencing has been widely applied to the detection and identification of gut microorganisms in insects. Gut bacteria in many phytophagous insects, including termites [10, 11], *Rhynchophorus ferrugineus* (palm weevil) [12] and *Dendroctonus armandi* (Chinese white pine beetle) [13] have been identified by 16S rRNA sequencing and show that the symbiotic bacteria primarily belong to *Escherichia* sp., *Serratia* sp., *Pantoea* sp., *Acinetobacter* sp., *Salmonella* sp., *Pseudomonas* sp., *Shigella* sp., *Staphylococcus* sp., *Klebsiella* sp., *Enterobacter* sp., *Erwinia* sp., *Vibrio* sp., *Proteus* sp. and *Bacillus* sp. Of these identified bacteria, *Serratia* sp. [14], *Pseudomonas* sp. [15] and *Bacillus* sp. [16] exhibited lignocellulose degradation activity.

The bamboo snout beetle, *Cyrtotrachelus buqueti*, is a serious pest of bamboo species, which include *Phyllostachys pubescens*, *Neosinocalamus affinis*, *Bambusa textilis* and *Dendrocalamus farinosus* [17]. *Cyrtotrachelus buqueti* also exhibits high lignocellulolytic enzyme activity [18]. However, to date, no studies have investigated the role of snout beetle gut microbiota in lignocellulose degradation, as has been done for termites [8] and other species of beetles [4–7]. The present study investigates the role of adult and larval *C*. *buqueti* gut bacteria using 16sRNA sequencing. Adult and larval *C*. *buqueti* were shown to carry similar loadings of lignocellulose-degrading bacteria. Furthermore, the degradation of bamboo shoots (*Bambusa emeiensis*) by snout beetle gut microbiota was investigated in vitro, and the results revealed that the microbiota of both adult and larval *C*. *buqueti* has lignocellulose-degrading ability.

## Results

### Gut structure in different age groups and microbial colonisation of the gut paunch

In insects, the gut is divided into foregut, midgut, and hindgut [19]. Although the foregut is mostly involved in mechanical treatment [20], delignification occurs in the midgut [21] and symbiotic microorganisms exist mainly in the hindgut, where the biomass is degraded [19].

**Fig. 1:**
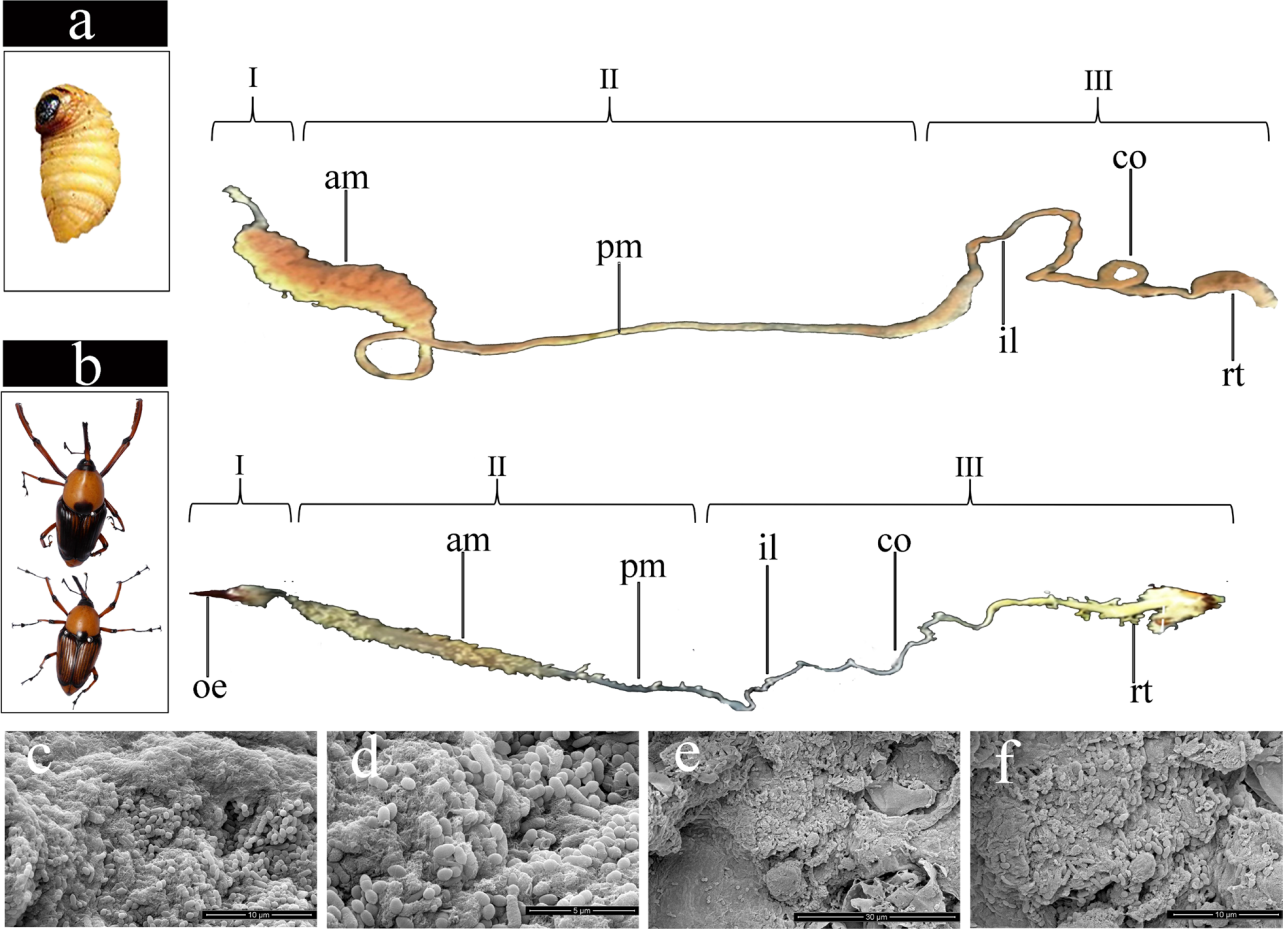
Intestinal tract structure in adult and larvae of *C*. *buqueti*. Morphological characteristics of larvae (**a**) and adults (**b**). I: foregut; II: midgut; III: hindgut; oe: oesophagus; am: anterior midgut; pm: posterior midgut; il: ileum; Co: colon; rt: rectum; Scanning electron micrographs global (**c**) and detailed (**d**) views of the adult intestinal tract. Scanning electron micrographs global (**e**) and detailed (**f**) views of the larvae intestinal tract

In the present study, the intestinal tract of the *C*. *buqueti* was analysed. Results showed that it comprises a foregut, a large midgut comprising an anterior and a posterior midgut, and a hindgut comprising a paunch, an ileum and a colon (Fig. 1a, b). The total length of the larval intestinal tract was 21.25 ± 0.55 cm, which corresponds to 3.8 times its body length. The foregut represented 3.33–3.7% of the total length, whereas the midgut represented 52–58%, and the hindgut represented 38–44%. On the other hand, the total length of the adult intestinal tract was 9.83 ± 0.84 cm, which corresponds to 2.4 times of its body length. The foregut represented 13–20% of the total length, the midgut represented 28–32%, and the hindgut represented 51–55%.

The distribution of microbial cells colonising the gut paunch was analysed by SEM (Fig. 1c–f). In adults and larvae, the gut paunch was mostly inhabited by rods and cocci, indicating the existence of symbiotic microbiota in the gut of adult and larval *C*. *buqueti*.

### SEM, BSPs components and culture reducing sugar content after treatment

In insects that digest lignocellulose, gut symbiotic microbes are ubiquitous and involved in lignocellulose degradation in various degrees [4]. To investigate the degradation efficiency of the gut symbiotic microbes in *C*. *buqueti*, the gut symbiotic microbiota of female (CCJ) and male (XCJ) adults and larvae (YCJ) beetles was extracted and used for in vitro degradation of bamboo shoot particles (BSPs) for 6 days.

**Fig. 2:**
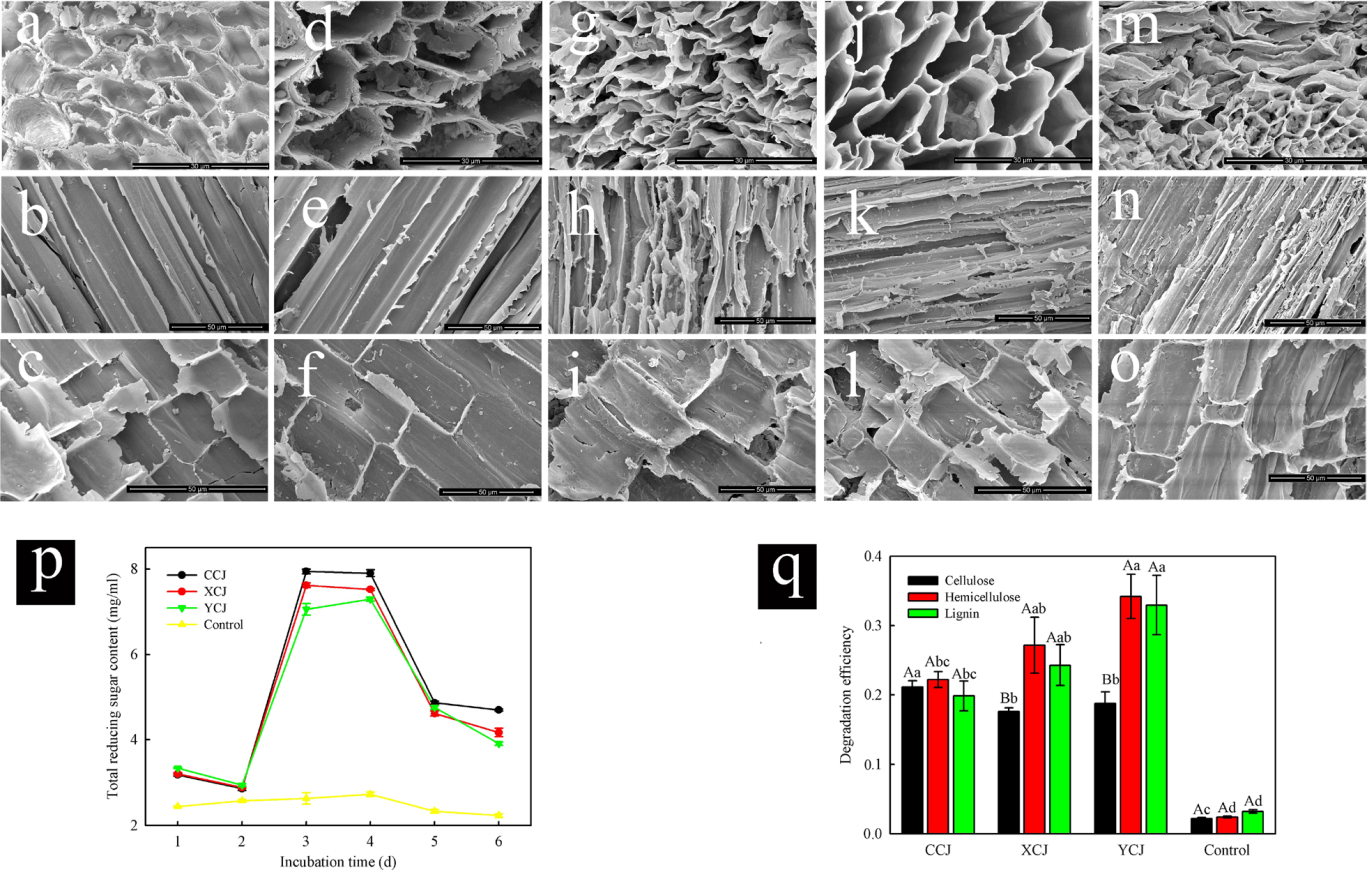
Scanning electron microscopy (SEM), bamboo shoot particles (BSPs) components and reducing sugar content in cultures after in vitro treatment. **a**–**c** SEM for raw BSPs; **d**–**f** SEM for BSPs in the control group; **g**–**i** SEM for BSPs after 72 h of treatment in CCJ; **j**–**l** SEM for BSPs after 72 h of treatment in XCJ; **m**–**o** SEM for BSPs after 72 h of treatment in YCJ; **p** amount of reducing sugar in the culture after 6 days of treatment; **q** cellulose, hemicellulose and lignin degradation efficiency of BSPs after 6 days of treatment. CCJ: gut symbiotic microbiota of female; XCJ: gut symbiotic microbiota of male; YCJ: gut symbiotic microbiota of larvae. Descriptive data were expressed as mean ± Standard Error of Mean; the different normal letters indicated significant difference at different developmental stage at 0.05 level (*n* = 3); the different capital letters indicated significant difference in different components at 0.05 level (*n* = 3)

Changes in the fibre microstructure of plant raw materials can be accurately observed using SEM. Following in vitro exposure to the intestinal tract microbiota of female, male, and larval beetles, the BSPs were observed using SEM. The results showed that the surface of lignocellulose from untreated, control BSPs was rough, showing gully-like surface features and a dense structure (Fig. 2a–f). However, no significant differences were observed between raw (Fig. 2a–c) and control (Fig. 2d–f) samples. On the other hand, after 72 h of treatment, the cell walls of BSPs became thinner with an enlarged cellulosic cavity (Fig. 2g, j, m) and many cracks appeared on the surface (Fig. 2h, k, n). Moreover, compared to what is shown in Fig. 2c (Fig. 2i, l, o) new dimples or holes were found on the surface of BSPs. These results are consistent with previous reports [22, 23]. A comparison between the pre- and post-treatment microstructure of BSPs revealed that its structure was significantly destroyed by treatment.

Since the reducing sugars in the culture medium was mainly derived from the hydrolysis of cellulose and hemicellulose in BSPs, the reducing sugar content was determined to reflect the degree of conversion of lignocellulose. The results showed small content changes during the first day of digestion, which increased henceforth. The maximum value was reached on day three in adults and on day 4 in larvae, subsequently decreasing until day 6 (Fig. 2p). The increase of reducing sugars indicated the degradation of cellulose and hemicellulose [24].

We then determined the degradation efficiencies of lignocellulose from BSPs after 6 days of treatment. As shown in Fig. 2q, it was found that cellulose, lignin, and hemicellulose were partially removed by the gut symbiotic microbiota. The degradation efficiencies of CCJ, XCJ, and YCJ digestions were: 21.11%, 17.58% and 18.74%, respectively, for cellulose; 22.22%, 27.18% and 34.20%, respectively, for hemicellulose and 19.83%, 24.30% and 32.97%, respectively, for lignin (Fig. 2q). These results showed that gut symbiotic microbiota degrading cellulose, lignin, and hemicellulose in BSPs could be cultured in vitro and then applied to the pretreatment and hydrolysis of bamboo lignocellulose.

### Determination of lignocellulolytic enzyme activity

**Fig. 3:**
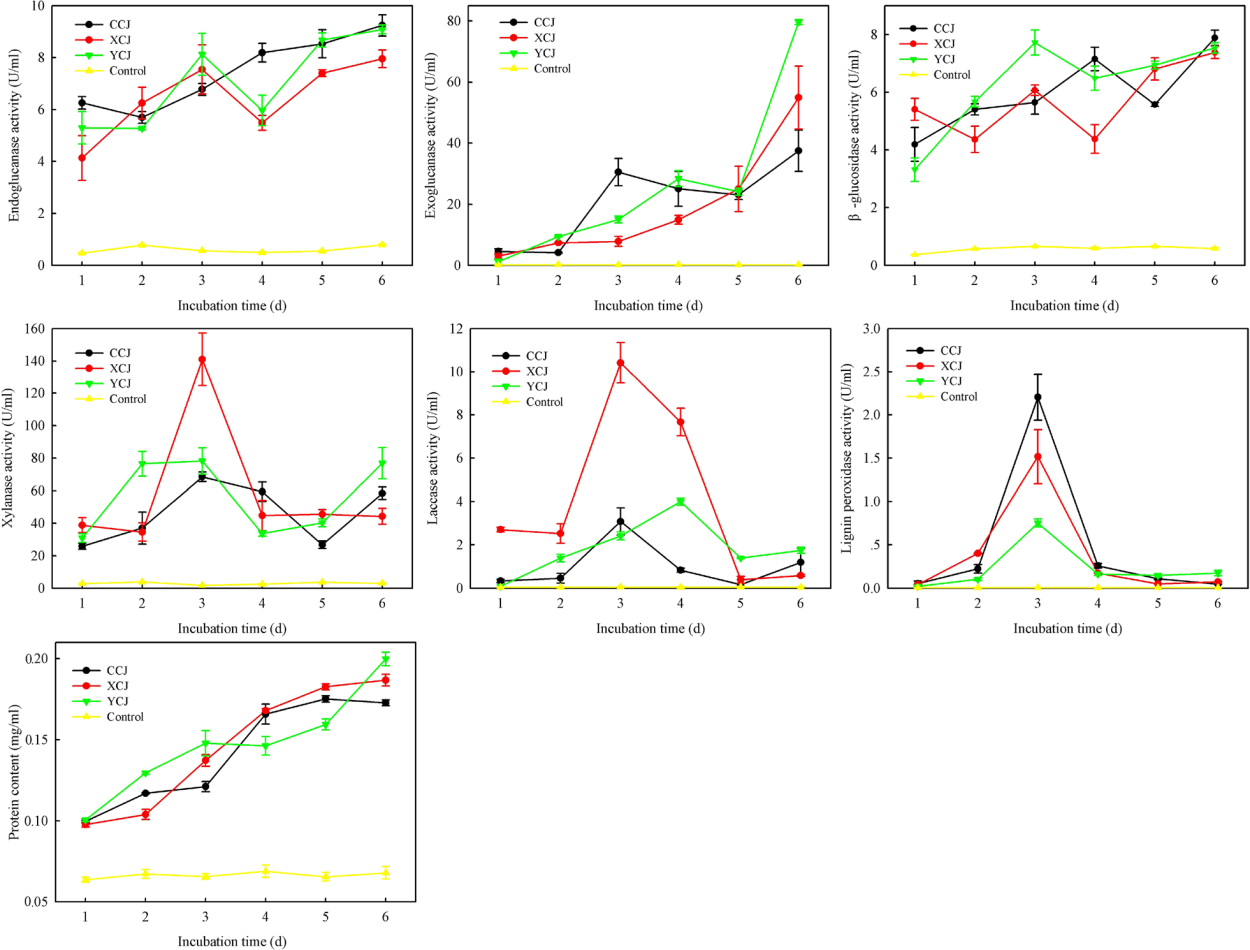
The lignocellulolytic enzyme activities of *C*. *buqueti* displayed by gut symbiotic microbiota 1, 2, 3, 4, 5 and 6 days after being co-cultured with BSP in vitro

To investigate the mechanism of the degradation of BSPs lignocellulose in vitro, the activities of the lignocellulolytic enzymes present in the gut symbiotic microbiota of adults and larvae, such as endoglucanase, β-glucosidase, xylanase, exoglucanase, laccase and lignin peroxidase, were determined. As presented in Fig. 3, the activity of cellulase, including endoglucanase, β-glucosidase and exoglucanase increased with treatment, whereas that of xylanase, laccase and lignin peroxidase first increased and subsequently decreased. To investigate whether the higher enzyme activity was caused by an increase of protein secretion, we determined the secreted protein content during the course of the treatment. The amount of secreted proteins was found to increase continuously (Fig. 3), indicating that the higher enzyme activity was partially caused by increased protein secretion.

### Gas chromatography–mass spectrometry (GC–MS) of lignin degradation products of BSP in vitro

GC–MS has been shown to effectively detect lignin degradation products [24]. We used GC–MS to identify the type of low-molecular-weight products obtained from the cultures after six days of treatment. The total ion chromatograms (TIC) of compounds extracted from control and treated samples revealed that several small molecular aromatic metabolites occurred only in treated samples. Such was the case of phenol (RT 7.22), phenylethyl alcohol (RT 10.71), 2-methoxyphenol (RT 9.85), 4-ethylphenol (RT 12.36) and p-cresol (RT 9.97), which are considered as the basic units of the lignin polymer (Table 1; Additional file 1: Figure S1). This result indicates that lignin in BSPs was degraded to produce phenolic compounds, with the guaiacyl structure unit as the main body. Furthermore, bond breakage between the methoxyl groups linked to the benzene ring of guaiacyl produces one-substituted aromatics, such as phenol and dimethyl phenol.

**Table 1:** Identification of metabolites as trimethylchlorosilane (TMS) derivatives from BSP samples

Retention time	Compound	Control^a^	Treated^b^	Treated^c^	Treated^d^
4.33	4-Benzyloxy-3-methoxy-2-*n*itro-benzaldehyde	−	−	−	+
4.90	Phenyl-butanedioic acid	−	−	−	+
7.22	Phenol	−	+	+	+
7.49	Succinic acid	−	−	+	−
9.85	2-Methoxy-phenol	−	+	+	+
9.97	*p*-Cresol	−	−	+	−
10.71	Phenylethyl alcohol	−	−	+	−
12.36	4-Ethyl-phenol	−	+	−	−
15.08	4-Ethyl-2-methoxy-phenol	−	−	+	+

^a^Non-inoculated (control) BSP samples

^b^CCJ degraded BSP samples

^c^XCJ degraded BSP samples

^d^YCJ degraded BSP samples

Bamboo lignin contains guaiacyl (G), syringyl (S) and *p*-hydroxyphenyl (H) units, belonging to the G–S–H type [25]. It was previously reported that most bamboo cell walls are mainly composed of guaiacyl units at the early stage of lignification [26]. In the present study, BSPs were degraded to produce phenolic compounds; with guaiacyl structure unit as the main body, indicating that the G unit of BSPs was degraded by the beetles’ gut symbiotic microbiota.

### Structure of bacterial communities

To investigate the bacterial diversity in the larval and adult *C*. *buqueti* guts, a bacterial 16S rRNA gene clone library was constructed by PCR. The whole DNA was extracted from the whole intestinal tracts of the adults and larvae. The larval and adult guts were labelled as ‘YWG’ and ‘AWG’, respectively. A total of 1,957,125 raw reads from 15 YWGs and 15 AWGs were characterised by Illumina high-throughput sequencing. After quality trimming, 1,889,645 high-quality clean tags were obtained and binned into 31,196 operational taxonomic units (OTUs) (Table 2).

**Table 2:** Estimated richness and diversity of bacterial communities in the gut of adult and larval *Cyrtotrachelus buqueti* obtained from pyrosequencing analysis

Sample	Number of reads	Number of OTUs	Species richness		Goods coverage	Community diversity		PD whole tree
			ACE	Chao1		Shannon	Simpson	
AWG1	31,150	182	278.8226	289.2500	0.9977	0.6883	2.4760	9.3095
AWG2	56,943	859	1066.7443	1049.8792	0.9948	0.8003	3.7530	22.9572
AWG3	60,119	923	1233.6556	1244.2368	0.9935	0.7983	3.6131	22.7947
AWG4	52,573	1101	1330.6848	1321.6359	0.9932	0.8031	3.8502	27.3003
AWG5	65,985	974	1282.3685	1264.5150	0.9941	0.6436	3.1327	24.3539
AWG6	37,952	841	1203.8728	1144.7186	0.9896	0.6455	3.3916	21.7401
AWG7	50,506	1059	1384.7723	1364.1053	0.9919	0.7939	4.0728	26.7679
AWG8	62,913	987	1200.7457	1172.2312	0.9945	0.4227	2.5382	26.0306
AWG9	62,582	733	1080.6907	1077.8824	0.9945	0.8028	3.4679	20.8508
AWG10	43,560	876	1302.5937	1272.2903	0.9906	0.7623	3.6795	24.8618
AWG11	60,729	1097	1453.0054	1438.9048	0.9927	0.6030	3.5085	33.2195
AWG12	48,588	910	1312.9882	1270.3208	0.9909	0.7812	3.9470	24.6801
AWG13	57,739	930	1240.5902	1321.8898	0.9927	0.7341	3.6324	25.3036
AWG14	66,286	853	1326.6953	1390.7027	0.9937	0.7559	3.2992	26.4275
AWG15	30,357	470	625.4256	602.0000	0.9935	0.7590	3.6334	14.5617
Mean of YWG	52,532	853	1154.9104	1148.3042	0.9932	0.7196	3.4664	23.4106
YWG1	89,632	1290	1529.4723	1562.8947	0.9957	0.8009	4.2393	29.2471
YWG2	73,877	1234	1510.1208	1567.0368	0.9948	0.7527	3.6564	27.8022
YWG3	85,232	1328	1561.7405	1618.0491	0.9958	0.7966	4.1156	30.2476
YWG4	64,352	999	1194.9094	1244.0476	0.9957	0.6055	2.9833	22.9895
YWG5	75,328	1079	1407.4523	1419.0066	0.9953	0.4898	3.0009	26.8487
YWG6	80,143	989	1275.2326	1275.1728	0.9954	0.5682	3.0797	25.9651
YWG7	106,571	1410	1630.6420	1665.7977	0.9966	0.7790	4.0954	30.8279
YWG8	91,679	1518	1955.5009	1987.6239	0.9941	0.6591	3.7524	34.5241
YWG9	95,196	1416	1691.5796	1732.2813	0.9952	0.8102	4.3674	31.9566
YWG10	71,581	1405	1871.9408	1866.4194	0.9926	0.8188	4.6616	30.3750
YWG11	65,622	1129	1637.4775	1608.9306	0.9930	0.6933	3.9235	27.3060
YWG12	66,211	1097	1457.2757	1471.6908	0.9940	0.7594	3.9203	25.9297
YWG13	59,952	1312	1730.7135	1699.8066	0.9917	0.8682	5.0228	32.0469
YWG14	61,949	987	1218.5840	1319.5909	0.9944	0.7178	3.9469	23.7491
YWG15	81,818	1208	1508.3660	1559.4024	0.9944	0.8290	4.5729	27.4453
Mean of YWG	77,942	1226	1545.4005	1573.1834	0.9946	0.7299	3.95589	28.4841
P value	9.2251E−06	4.1182E−05	4.4533E−04	1.4405E−04	3.2404E−02	7.9292E−01	1.8278E−02	4.8929E−03

OTU: operational taxonomic units; PD: phylogenetic diversity

**Fig. 4:**
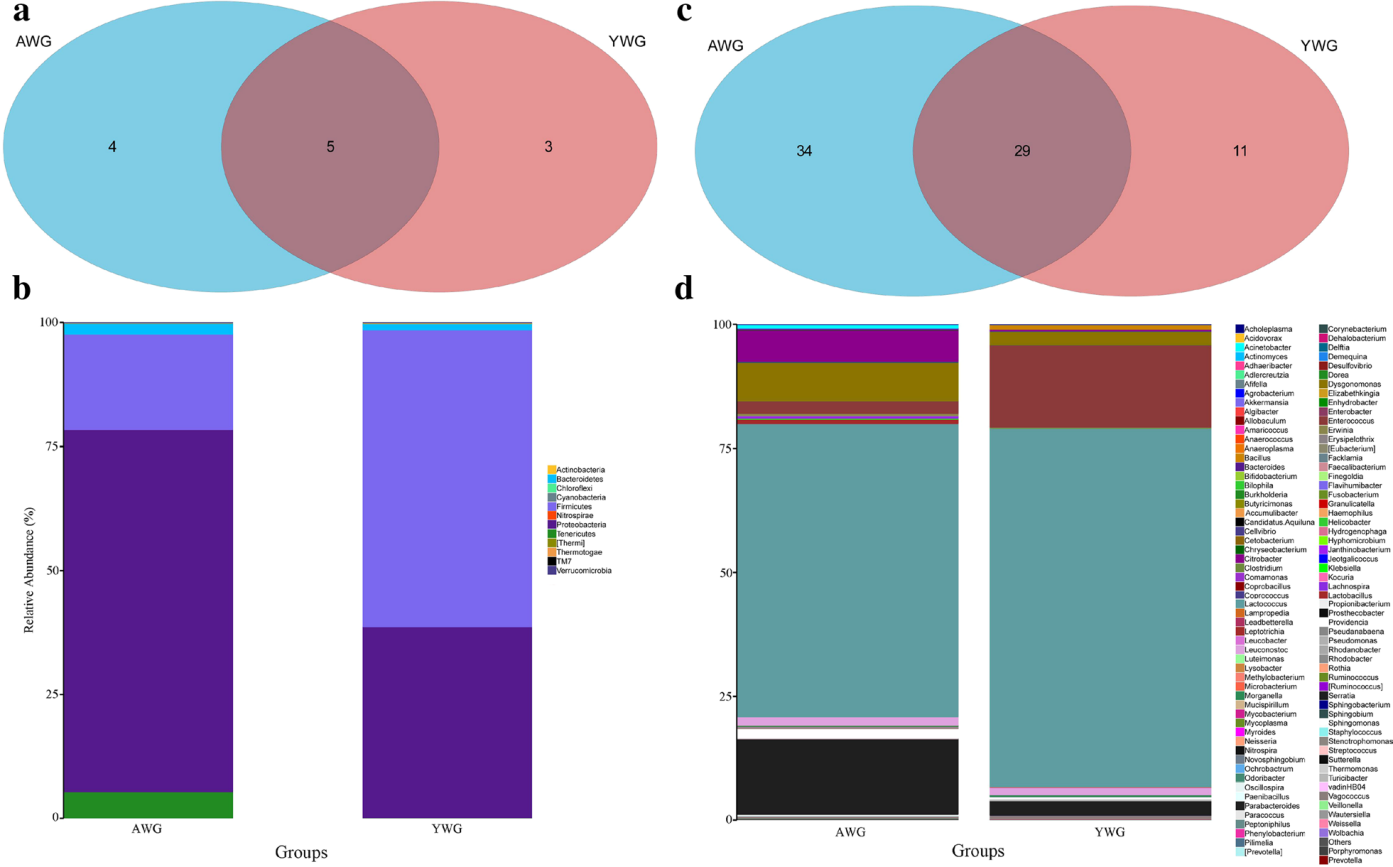
Bacterial operational taxonomic units (OTUs) composition in adult and larvae beetles (AWG and YWG). **a** Venn diagram showing the coincidence of phyla between the groups. **b** Phylum-level comparison of bacterial OTUs between the groups. **c** Venn diagram showing the overlap of genera between groups. **d** Genus-level comparison of bacterial OTUs between the groups

At the phylum level, a total of 14 prokaryotic phyla were identified, among which five were common to both groups (Fig. 4a, b). In both groups, Proteobacteria (38.6–73.0%) and Firmicutes (19.2–59.8%) were the most abundant microbial communities. The two most increased phyla in the AWG group were Proteobacteria and Tenericutes; which, compared with the YWG group, had a relative abundance increase of 89% and 3095%, respectively. On the contrary, two phyla, Firmicutes and Fibrobacteres, decreased by 63% and 68%, respectively (Additional file 2: Table S1). At the genus level, besides unclassified OTUs, 74 genera were detected, among which 29 were common to both groups (Fig. 4c, d). The abundance of all genera in each group is shown in Additional file 3: Table S2. *Lactococcus* (59.1–72.3%) was consistently abundant in both groups. Thirty-four genera were detected only in the AWG group, whereas 11 were detected only in the YWG group. Non-metric multi-dimensional scaling (NMDS) plots and similarity analysis (ANOSIM) (*p* < 0.05) (Additional file 4: Figure S2) revealed a divergence of the community structure in the AWG and YWG groups.

### Diversity and richness of microbial communities

**Fig. 5:**
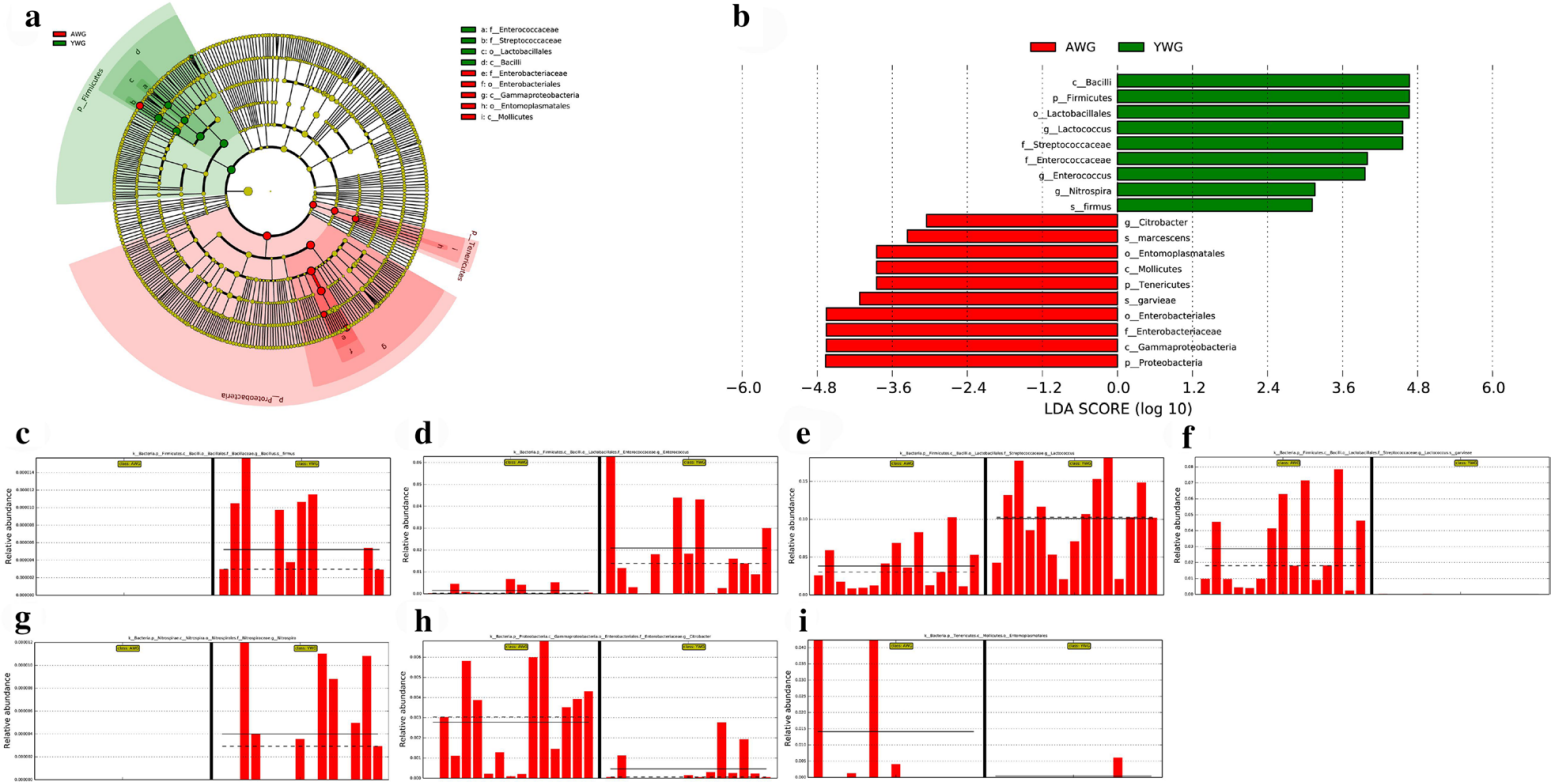
LEfSe analysis of the two groups. **a** Cladogram of the two groups. **b** LDA score histogram. The relative abundance of **c** *Bacillus firmus*, **d** *Enterococcus*, **e** *Lactococcus*, **f** *Lactococcus garvieae*, **g** *Nitrospira*, **h** *Citrobacter* and **i** Entomoplasmatales. Solid and dashed lines indicate mean and median, respectively. The *x*-axis in **c**–**i** represented the samples: AWG1-15 and YWG1-15

The YWG had the highest species richness (Chao 1, ACE and OTUs) (Table 2). A significant difference was observed between the groups regarding the diversity of microbial communities as determined by the Shannon index (*p* < 0.05), whereas the Simpson index revealed no significant difference (Table 2; Additional file 5: Figure S3). Differentially represented OTUs were analysed via linear discriminant analysis (LDA) effect size (LEfSe), a statistical measure used in metagenomic biomarker discovery (Fig. 5a, b). Genera such as *Lactococcus*, *Enterococcus* and *Nitrospira* and only one species—the *Bacillus firmus*—increased in YWG, whereas one genus and two species increased in AWG (Fig. 5c–i).

**Fig. 6:**
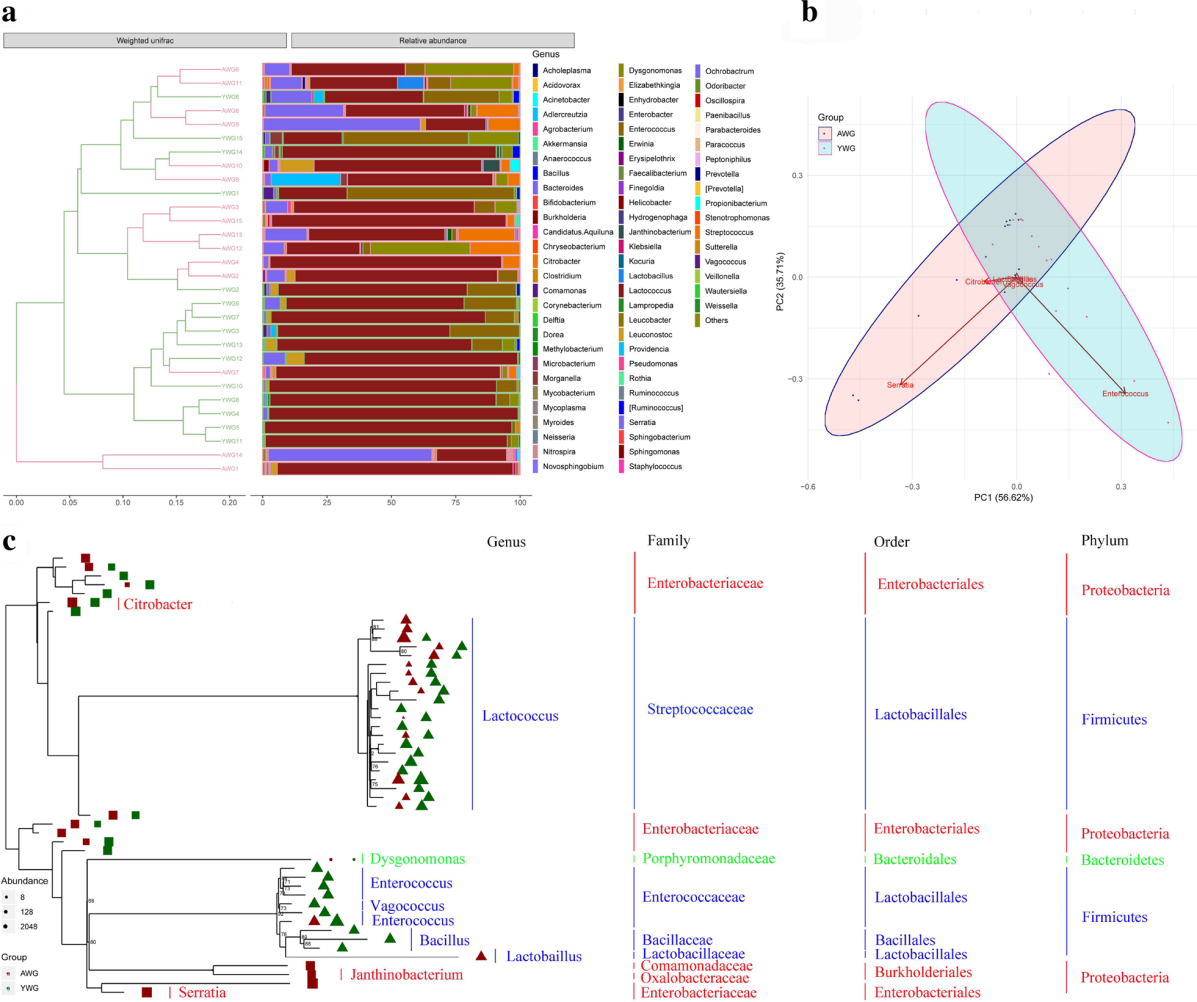
**a** Weighted UPGMA tree. **b** Beta diversity of the samples. **c** Maximum likelihood tree of 50 detectable OTUs (the relative abundance > 1% in the given sample). The complete 16S rRNA gene sequences of the corresponding species in the RDP database were used to construct the tree. Red indicates the OTUs in the AWG group, and green indicates the OTUs in the YWG group. Only the OTUs with significant differences (*p* < 0.05) in relative abundance are shown behind the branches. The size of the symbol indicates the relative abundance of OTUs

UniFrac analysis was performed to compare the degree of phylogenetic overlap in the microbial communities of AWG and YWG (Fig. 6a). Moreover, a total of six OTUs with maximum abundance, including *Lactococcus*, *Enterococcus*, *Bacillus*, *Citrobacter*, *Vagococcus* and *Serratia*, were used for principal component analysis and clearly separated into two groups. Specifically, the difference of PC1 was caused mostly by *Enterococcus* and *Serratia*, which were positively correlated. Additionally, the *Enterococcus* was positively correlated with the other OTUs except *Citrobacter*, whereas the *Serratia* was positively correlated with all OTUs (Fig. 6b). Maximum likelihood (ML) analysis of the 50 detectable OTUs showed that the significantly increased OTUs in the YWG group belonged to the families: Enterobacteriaceae, Streptococcaceae, Enterococcaceae, and Bacillaceae subdivision 4 (Fig. 6c).

### Functional analysis of microbial communities

**Fig. 7:**
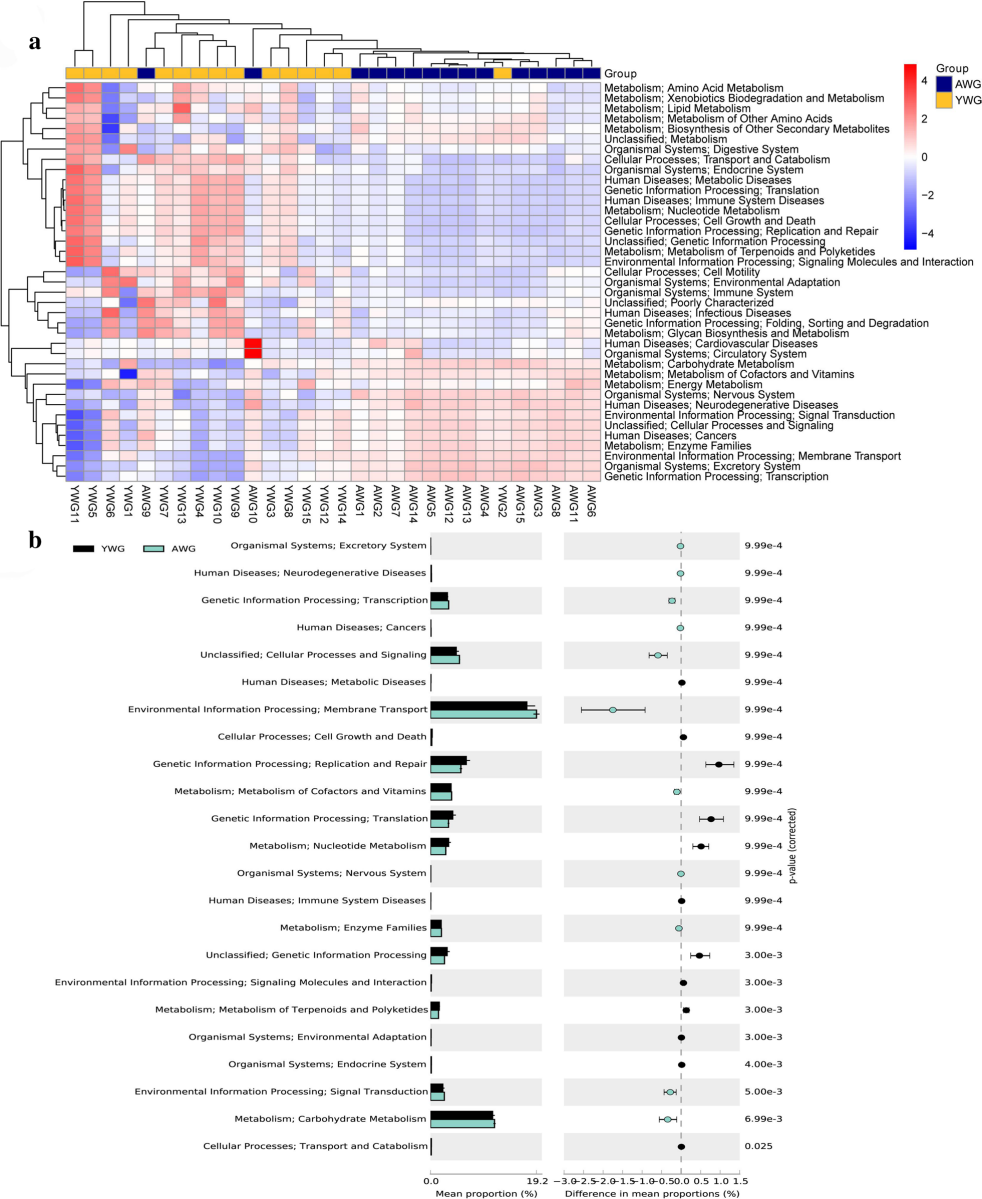
KEGG pathways enriched in adults and larvae of insects (AWG and YWG). **a** Cluster of pathways in AWG and YWG. **b** The relative pathways abundance was compared between AWG and YWG

In this study, PICRUSt was used to predict the microbial community metagenome [27]. At the KEGG L2 level, in AWG, OTUs were mainly enriched in metabolic pathways, such as amino acid metabolism, lipid metabolism, xenobiotics biodegradation and metabolism and human diseases. In YWG, the OTUs were enriched in energy metabolism, environment adaption, nucleotide metabolism and molecular signalling and interaction (Fig. 7a). The functions of each bacterial community in AWG and YWG were significantly different for all pathways (Fig. 7b).

Moreover, PICRUSts predicted an enrichment of the relative abundance of genes relevant to lignocellulose degradation, genes encoding the carbohydrate-active enzymes (CAZymes), including glycoside hydrolases (GHs), glycosyl transferases (GTs), polysaccharide lyases (PLs), carbohydrate esterases (CEs), and auxiliary activities (AAs) (Additional file 6: Figure S4). Among the CAZyme family genes included on the prediction, many were closely related to lignocellulose degradation.

### Composition and lignocellulose degradation potential of the core degradative microbiota

16sRNA-sequencing showed that *Lactococcus*, *Serratia*, *Dysgonomonas*, and *Enterococcus* represented about 84–94% of the microbiota, comprising the core digestive microbiota of AWG and YWG (Additional file 7: Figure S5). To further explore the potential role of *Lactococcus*, *Serratia*, *Dysgonomonas*, and *Enterococcus* in lignocellulose degradation, we compared their CAZymes content with that of *Cytophaga hutchinsonii* ATCC 33406, a model bacterium for lignocellulose bioconversion [28]. This comparison was based on the public CAZy databases (Table 3; Additional file 8: Table S3). Since no *Dysgonomonas* genome was available in the CAZy database, we manually annotated the CAZyme-coding sequences in the genome of the genus available to date in the NCBI Genome, i.e., that of *Dysgonomonas mossii* DSM 22836, *Dysgonomonas capnocytophagoides* DSM 22835, *Dysgonomonas macrotermitis* and *Dysgonomonas gadei* ATCC BAA-286. Compared with the genome of *C*. *hutchinsonii* ATCC 33406, the genomes of *Dysgonomonas* contained more GHs and CEs (Table 3; Additional file 8: Table S3). To evaluate the ability of *Lactococcus* to degrade lignocellulose in the intestine, we examined the CAZyme content of the 45 *Lactococcus* genomes currently available in the CAZy database (Additional file 8: Table S3). The 45 genomes present lower numbers of CAZyme than the *C*. *hutchinsonii* genome (Table 3; Additional file 8: Table S3). However, the examined *Lactococcus* genomes were mostly enriched in GH1 and GH13, which putatively exhibit (among others) β-glucosidase and β-galactosidase activity [29] and α-amylase [30], respectively (Additional file 8: Table S3). For *Serratia*, 72 genomes were examined, which showed a higher capacity to degrade carbohydrates, including lignocellulose, than *C*. *hutchinsonii*, due to a higher number of CAZyme families and genes in the genome (Table 3; Additional file 8: Table S3). Moreover, the GHs of *Serratia* were enriched in GH1, GH2, GH3, GH4, GH13, GH18 and GH23, and more AAs were performed. Eighty-six *Enterococcus* genomes were compared with the *C*. *hutchinsonii* genome, which exhibited more families and number, especially in GHs (Table 3; Additional file 8: Table S3). The examined *Enterococcus* genomes were mostly enriched in GH1, GH13 and GH73, which putatively exhibit (among others) β-glucosidase and β-galactosidase activity [22], α-amylase [23] and lysozyme [31], respectively (Additional file 8: Table S3).

**Table 3:** Total number of putative CAZy genes, including glycoside hydrolase (GH), carbohydrate esterase (CE), auxiliary activity (AA) and polysaccharide lyase (PL) genes in selected genomes

	GH^a^	CE^a^	AA^a^	PL^a^
45 *Lactococcus* genomes	8–23^b^ 9–51^c^	0–3 0–6	0–1 0–2	0–1 0–2
72 *Serratia* genomes	2–27 3–90	0–5 0–6	0–2 0–4	0–6 0–12
86 *Enterococcus* genomes	15–34 28–121	1–6 1–11	0–1 0–8	0–3 0–4
4 *Dysgonomonas* genomes	43–55 131–223	7–8 23–28	0–0 0–0	0–3 0–6
*C. hutchinsonii* ATCC 33406	19 50	7 14	0 0	3 3

This table also contains total number of CAZy genes number in selected genomes for the four genera of the core digestive microbiota of the insect and of *Cytophaga hutchinsoni*i ATCC 33406, a model bacterium for cellulose degradation [21]. For more details on the CAZyme content, see Additional file 7: Table S2

*C. hutchinsonii Cytophaga hutchinsonii*. Data retrieved from the CAZy databases (http://www.cazy.org)

^a^CAZyme class

^b^Total number of CAZyme families in the given class

^c^Total number of CAZyme genes in the given class

### Microbiota phenotype prediction using BugBase

**Fig. 8:**
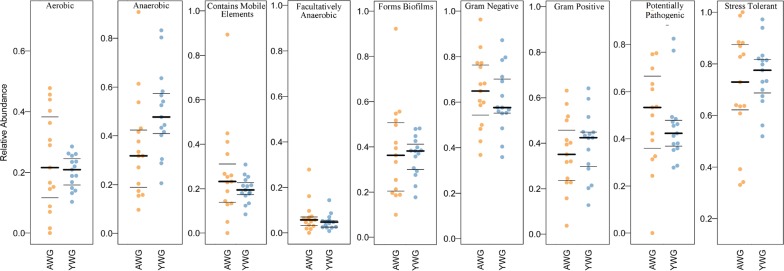
BugBase analyses, based on the NGS dataset. The outcome is grouped according to the modules AWG and YWG (*X*-axis). The relative abundance is given on the *Y*-axis. ‘Mobile elements’ refers to bacteria, most probably carrying mobile elements

BugBase (https://bugbase.cs.umn.edu/) is an algorithm that predicts the organism-level coverage of functional pathways; as well as biologically interpretable phenotypes, such as oxygen tolerance, Gram staining, and pathogenic potential within complex microbiota using either whole-genome shotgun or marker gene sequencing data [32]. However, no significant differences were observed in the relative abundance of all classifications (Fig. 8; Additional file 9: Table S4), indicating similarities in the bacterial phenotype of AWG and YWG. To date, few reports have focused on degradation by microbes, such as *Coriolus versicolor* [33] and *Galactomyces* sp. CCZU11-1 [34]. These predicted results will, therefore, expand the current knowledge on gut symbiotic microbiota of the beetle and support the culture of lignocelluloses-degrading bacteria in vitro.

## Discussion

The symbiotic microbiota of insects includes bacteria, protozoa, fungus and archaea, among which bacteria are the most representative. Symbiotic bacteria exist in Coleoptera, Blattaria, Isoptera, Diptera, Hemiptera, Mallophaga, and Anoplura [35] and the symbiotic bacteria of insects primarily belong to the phyla: Proteobacteria, Bacteroidetes, Firmicutes, Actinomycetes, Spirochetes, and Verrucomicrobia [36].

Gut symbiotic microbes play an important role in nutrient supply, digestion and absorption. Bashir et al. [37] isolated 42 cellulose-degrading bacteria from the gut of termites, pill bugs, and stem borers. Dantur et al. [38] isolated 118 cellulose-degrading bacteria from the larval intestine of *Diatraea saccharalis*, and Manfredi et al. [39] isolated 233 cellulose-degrading bacteria from *Spodoptera frugiperda* and *Diatraea saccharalis*, among which *Bacillus* and *Paenibacillus* were the most prevalent. Peterson et al. [40] used antibiotics to clarify the role of symbiotic bacteria on cellulose degradation by *Reticulitermes flavipes*. Furthermore, Shotorkhoft et al. [41] isolated three bacteria exhibiting ligninase activity from *Microcerotermes diversus* and discussed their ability to degrade wheat straw.

In the present study, we used 16sRNA sequencing to identify gut symbiotic bacteria in *C*. *buqueti*. Results showed that *Lactococcus*, *Serratia*, *Dysgonomonas*, and *Enterococcus* represented approximately 84–94% of the microbiota. Robert et al. [42] found that the most common cellulolytic strains isolated corresponded to the *Ruminococcus* and *Enterococcus* species found in the human colon. Shil et al. [43] showed that *Enterococcus* species participates in lignocellulose degradation by insects. In the present study, BSPs were treated with gut symbiotic microbiota, which revealed that the degradation efficiency of CCJ, XCJ and YCJ was 21.11%, 17.58% and 18.74%, respectively, for cellulose; 22.22%, 27.18% and 34.20%, respectively, for hemicellulose; and 19.83%, 24.30% and 32.97%, respectively, for lignin, indicating that symbiotic s degrade the cellulose, hemicellulose, and lignin of BSPs. Moreover, gut symbiotic microbiota has the potential to be cultured in vitro and at large scale; and then applied to the pretreatment and hydrolysis of bamboo lignocellulose. BugBase (https://bugbase.cs.umn.edu/) was used to predict microbiota phenotypes, which allowed the expansion of current knowledge regarding gut symbiotic microbiota of *C*. *buqueti*, with the potential to support in vitro cultures of lignocellulose-degrading bacteria.

## Conclusions

The present study investigated the bamboo lignocellulose-degrading ability of gut symbiotic microbiota of *C*. *buqueti*. The in vitro hydrolysis assay revealed bamboo lignocellulose-degrading efficiencies in CCJ, XCJ, and YCJ of 21.11%, 17.58% and 18.74% for cellulose; 22.22%, 27.18% and 34.20% for hemicellulose, and 19.83%, 24.30% and 32.97% for lignin. Results from the 16sRNA-sequence identified four microbes, namely *Lactococcus*, *Serratia*, *Dysgonomonas* and *Enterococcus* representing approximately 84–94% of the microbiota in this system. These microorganisms exhibited abundant CAZyme genes and lignocellulose-degrading ability. Finally, predicted results of BugBase support the in vitro culturing of lignocellulose-degrading bacteria of the gut, such as *C*. *buqueti*. This study has enriched our knowledge of bamboo lignocellulose-degrading microbiota, which can be applied to bamboo lignocellulose bioconversion.

## Methods

### Collection of insect samples

Adult and larval *C*. *buqueti* were collected in August 2018 in the Muchuan City, Sichuan Province, China (N103°98′, E28°96′). All adults were collected at the 3rd day after emergence [44]. Intestines were extracted from 15 individual adults (males and females) and 15 individual larval beetles, and then immediately stored in liquid nitrogen to ensure gut microbiome stabilisation until subjection to DNA extraction and 16sRNA-sequencing.

### In vitro assay of bamboo shoot particles (BSPs) degradation by gut symbiotic microbiota

In the present study, BSPs at the early stage of lignification were used. BSPs were prepared by drying to a constant weight at 65 °C, crushing to particles using a pulverising machine, and straining through a 40-mesh sieve. Gut symbiotic microbiota of adult and larval beetles was prepared by grounding and mixing, respectively. The mixed suspension was then cultured in liquid medium 1 (pH 7.2) composed of 0.04 g yeast extract, 0.1 g malt extract, 2 g CaCO_3_ and 10 g BSPs for 15 d. Two millilitres of the culture was added into a second liquid medium (pH 7.2) composed of 0.5 g yeast extract, 0.5 g malt extract, 0.5 g tryptone, 0.5 g NaCl, 0.2 g KH_2_PO_4_, 0.13 g MgSO_4_·7H_2_O and 0.5 g CaCl_2_. The assay was then performed following the steps listed in Table 4.

**Table 4:** Design and determination methods of gut symbiotic microbiome degradation of bamboo shoot particles (BSPs) in vitro

	Experimental group	Control group	Incubation time	Temperature
Experimental design	2 mL culture + 98 mL pH 7.2 liquid medium 2^a^ + 5 g BSPs	100 mL pH 7.2 liquid medium 2^a^ + 5 g BSPs	6 days	37 °C

	Low-molecular-weight products	Lignocellulose	Surface structure of BSPs	Reducing sugar
Determination index and methods	GC–MS [17]	Van Soest method [38]	SEM	DNS [37]

^a^A liquid medium (pH 7.2) composed of 0.5-g yeast extract, 0.5-g malt extract, 0.5-g tryptone, 0.5-g NaCl, 0.2-g KH_2_PO_4_, 0.13-g MgSO_4_·7H_2_O and 0.5-g CaCl_2_

As shown in Table 4, the samples were placed into a 250-mL conical flask and incubated under constant-temperature shock at 37 °C and 150 rpm for 6 days. The reaction products were inactivated at 100 °C for 30 min and centrifuged at 13,000 rpm for 10 min, after which the supernatant was collected and dried at 65 °C to a constant weight. The dried deposit was weighed, and the levels of cellulose, hemicellulose, and lignin were determined and used for SEM. The supernatant was used to determine reducing sugar and low-molecular-weight products.

#### Determination of reducing sugar

Reducing sugar was identified using 3,5-dinitrosalicylic acid [45].

#### Determination of cellulose, hemicellulose and lignin

The cellulose, hemicellulose, and lignin contents of the dried BSPs were determined using the Van Soest method [46] and the following formulas:

$${\text{Hemicellulose}}\;{\text{content}} = {\text{neutral}}\;{\text{detergent}}\;{\text{fibre }}\left( {\text{NDF}} \right) - {\text{acid detergent fibre }}\left( {\text{ADF}} \right)$$

$${\text{Cellulose}}\;{\text{content}} = {\text{ADF}} - {\text{acid detergent lignin }}\left( {\text{ADL}} \right)$$

$${\text{Lignin content}} = {\text{ADL}} - {\text{ash}}\;{\text{content}}$$

#### Cellulose, hemicellulose and lignin degradation efficiencies

$${\text{Cellulose}}\;\;{\text{degradation}}\;\;{\text{efficiency }} = \left( {1 - \frac{{{\text{The}}\;{\text{mass}}\;{\text{of}}\;{\text{cellulose}}\;{\text{in}}\;{\text{deposit}} }}{{{\text{The}}\;{\text{mass}}\;{\text{of}}\;{\text{cellulose}}\;{\text{in}}\;{\text{raw}}\;{\text{material}}}}} \right) \times 100\%$$

$${\text{Hemicellulose}}\;{\text{degradation}}\;\;{\text{efficiency}} = \left( {1 - \frac{{{\text{The}}\;{\text{mass}}\;{\text{of}}\;{\text{hemicellulose}}\;{\text{in}}\;{\text{deposit}} }}{{{\text{The}}\;{\text{mass}}\;{\text{of}}\;{\text{hemicellulose}}\;{\text{in}}\;{\text{raw}}\;{\text{material}}}}} \right) \times 100\%$$

$${\text{Lignin}}\;{\text{degradation}}\;\;{\text{efficiency}} = \left( {1 - \frac{{{\text{The}}\;{\text{mass}}\;{\text{of}}\;{\text{lignin}}\;{\text{in}}\;{\text{deposit}} }}{{{\text{The}}\;{\text{mass}}\;{\text{of}}\;{\text{lignin}}\;{\text{in}}\;{\text{raw}}\;{\text{material}}}}} \right) \times 100\%$$

#### Scanning electron microscopy (SEM)

BSPs collected 72 h after treatment were analysed using SEM (Hitachi 3400N, Japan), to observe the samples’ surface morphology. Prior to imaging, the samples were sprayed with gold to a thickness of ~ 10 nm using the E-1010 sputtering film coating machine (Japan). The SEM operating current and voltage were 81 mA and 10 kV, respectively.

#### Determination the lignocellulolytic enzymes activities

From the co-cultures, 2-mL samples were withdrawn at 1, 2, 3, 4, 5 and 6 days for assays determining lignocellulolytic enzyme activity. Endoglucanase (EC 3.2.1.4), exoglucanase (EC 3.2.1.91), β-glucosidase (EC 3.2.1.21), lignin peroxidase (LiP)-like, laccase-like, and xylanase enzyme activities were analysed as previously described by Luo et al. [18]. Briefly, carboxymethyl cellulose (CMC), microcrystalline cellulose (MCC), salicin, veratryl alcohol (VA), 2,2′-azino-bis (ABTS) and xylan were used as substrates to determine endoglucanase, exoglucanase, β-glucosidase, LiP-like, laccase-like and xylanase, respectively. All assays were performed five times.

#### Gas chromatography–mass spectrometry (GC–MS)

On day 6, control and treatment cultures were collected and centrifuged at 8000 rpm for 20 min to remove biomass. The supernatants were collected and treated using the method described by Raj et al. [47]. Briefly, the supernatants were extracted three times using equal volumes of dichloromethane, collected extracted liquor, dewatered with anhydrous Na_2_SO_4_ and filtered and concentrated to ~ 1 mL. Next, 100-µL dioxane and 10-µL pyridine were added to the sample followed by silylation with 50-µL trimethyl silyl (*N*,*O*-bis[trimethylsilyl]trifluoroacetamide [BSTFA]/trimethylchlorosilane [TMS] = 99/1 [v/v]). Gas chromatography–mass spectrometry (GC–MS) was analysed according to the procedure described by Chen et al. [48]. The TMS were identified by comparing their mass spectra with the NIST library.

### DNA extraction, amplification and sequencing of 16S rRNA encoding gene sequences

DNA from different samples was extracted using the E.Z.N.A. ^®^Stool DNA Kit (D4015, Omega, Inc., USA) according to the manufacturer’s instructions. PCR amplification was performed in 25 μL of the following reaction mixture: 50-ng DNA, 12.5-μL Phusion Hot start flex 2X Master Mix (NEB, M0536L), 2.5-μL forward primer 338F 5′-ACTCCTACGGGAGGCAGCAG-3′ and 2.5-μL reverse primer 806R 5′-GGACTACHVGGGTWTCTAAT-3′ [49]. The PCR was performed at 98 °C for 30 s, 35 cycles of 98 °C for 10 s, 54 °C for 30 s, 72 °C for 45 s and 72 °C for 10 min. The amplicon pools were prepared for sequencing. The libraries were sequenced on Illumina MiSeq Instrument (Illumina Inc., San Diego, CA, USA) using the 300 bp paired-end protocol.

### Sequence analysis

QIIME v1.9.1 [50] was used to further analyse the sequences, using scripts available in the Qiime website (http://qiime.org/). First, potential chimeras were identified in valid sequences using de novo Uchime (usearch v9.0.2132_i86linux32) [51] and removed with Qiime build-in python scripts. The resulting sequences were defined as good sequences. The final good sequences were clustered into OTUs at 97% similarity using the de novo UCLUST algorithm [52]. Taxonomic assignment was then performed using the GreenGenes database (version 13.8) [53], after which an OTU abundance table was constructed using the Qiime python scripts. Multiple sequence alignment was conducted using the PyNAST (v1.2.2) [54] software, and a phylogenetic tree was constructed using FastTree (v2.1.9) [55], to investigate the phylogenetic relationship of different OTUs. To reduce the noise of amplicon datasets and make the data more credible, the OTUs whose abundance was lower than 0.001% of the total were removed, resulting in a modified OTU abundance table [56]. Using the phylogenetic tree and the modified OTU abundance table, alpha diversity (Ace, Chao1, Shannon, Simpson, Observed OTUs, goods_coverage, PD_whole tree) was calculated using the Qiime script, and beta diversity (Bray–Curtis, weighted and unweighted UniFrac) was estimated with the Phyloseq package (v1.20.0) [57]. Both biodiversities were visualised using the R programme (v3.4.1).

### Differentially abundant OTUs and microbes

DESeq 2 (v1.16.1) was used to detect differentially abundant OTUs among groups [58]. *P*_adj_ < 0.01 was considered as statistically significant. DESeq2 tests were performed using the Qiime python script. Differences among microbes regarding abundance of different taxonomic ranks were determined using a Metastats analysis, which is based on a non-parametric *t*-test [59]. The relative abundance of a microbe in a sample was calculated by determining the read count normalised by the total reads in that sample. Microbes with a relative abundance lower than 1% in all samples were classified into ‘others’. A level of *p* < 0.05 was considered as statistically significant. Metastats tests were performed using the EDDA R package (v1.10.0) [60].

### LEfSe analysis

The LEfSe algorithm was used to identify different abundance biomarkers between the two groups [61]. LEfSe couples robust tests such as Kruskal–Wallis test with quantitative tests such as Wilcoxon rank-sum test. After LDA, the features are ranked by effect size. An effect size threshold above 3 (on a log10 scale) was used for all biomarkers discussed in this study.

### Inferred metagenomics and phenotypes

The PICRUSt (v1.1.2) (http://picrust.github.io) was used to predict microbial community metagenome [27]. First, a collection of closed-reference OTUs was obtained from the filtered reads of QIIME v 1.9.1 and by querying the data against the GreenGenes database (http://greengenes.lbl.gov). The OTUs were assigned at 97% identity. PICRUSt was used to predict and derive relative KEGG pathway abundance. Microbial phenotypes were predicted using BugBase (https://bugbase.cs.umn.edu/) [32], a software that relies on the tools PICRUSt, IMG, KEGG and PATRIC.

### Statistical analysis

Statistical analyses were performed using SPSS 19.0 (IBM SPSS, Armonk, NY, USA). Descriptive data were expressed as mean ± standard error of mean (SEM). A Student *t*-test was used to compare the means from two groups. Comparisons of more than two groups were performed using analysis of variance. A level of *p* < 0.05 indicated a statistically significant difference.

## Additional files

**Additional file 1: Figure S1.** TIC of dichloromethane extract analysed as TMS derivative from control (A) and treatment CCJ (B), XCJ (C) and YCJ (D) in vitro. CCJ: gut symbiotic microbiota of female beetle; XCJ: gut symbiotic microbiota of male beetle; YCJ: gut symbiotic microbiota of beetle larvae.

**Additional file 2: Table S1.** Microbial composition of the AWG and YWG groups at the phyla level.

**Additional file 3: Table S2.** Microbial composition of the AWG and YWG groups at the genus level.

**Additional file 4: Figure S2.** Non-metric multidimensional scaling (NMDS) analysis of the Bray–Curtis similarity coefficients based on the relative abundance of OTUs in the given sample.

**Additional file 5: Figure S3.** Boxplot analysis comparing the bacterial OTUs between the two groups.

**Additional file 6: Figure S4.** Relative abundance of PICRUSt-predicted CAZyme genes relevant to lignocellulose degradation. (A) Glycoside hydrolases (GHs). (B) Glycosyl transferases (GTs). (C) Carbohydrate esterases (CEs). (D) Carbohydrate-binding modules (CBMs). (E) Auxiliary activities (AAs).

**Additional file 7: Figure S5.** Composition of the digestive core microbiota at the genus level.

**Additional file 8: Table S3.** Number of annotated genes for each GH, CE, PL and AA family in all *Lactococcus*, *Serratia*, *Enterococcus* and *Dysgonomonas* genomes available at http://www.cazy.org/and in *Cytophaga hutchinsonii* ATCC 33406. GH, glycoside hydrolase; CE, carbohydrate esterase; AA, auxiliary activity; PL, polysaccharide lyase; CAZyme, carbohydrate-active enzyme.

**Additional file 9: Table S4.** Relative abundance of the predicted classifications in each sample by BugBase.

## Abbreviations

*C. buqueti*: *Cyrtotrachelus buqueti*
KEGG: Kyoto Encyclopedia of Genes and Genomes
OTU: operational taxonomic units
LDA: linear discriminant analysis
SEM: scanning electron microscopy
GHs: glycoside hydrolases
GTs: glycosyltransferases
CEs: carbohydrate esterases
CBMs: carbohydrate-binding domains
PLs: polysaccharide lyases
AAs: auxiliary activities
CAZyme: carbohydrate-active enzymes
NCBI: The National Center for Biotechnology Information
ABTS: [2,2′-Azino-bis (3-ethylbenzothiazoline-6-sulfonic acid)]
BSPs: bamboo shoot particles
CCJ: gut symbiotic microbiota of female
XCJ: gut symbiotic microbiota of male
YCJ: gut symbiotic microbiota of larvae
LEfSe: Linear Discriminant Analysis Effect Size
TIC: total ion chromatograms
