# Supplementary figures and images for: Bamboo lignocellulose degradation by gut symbiotic microbiota of the bamboo snout beetle Cyrtotrachelus buqueti
Source: Biotechnol Biofuels. 2019 Apr 1;12:70. doi: 10.1186/s13068-019-1411-1 (PMC6442426; doi:10.1186/s13068-019-1411-1)

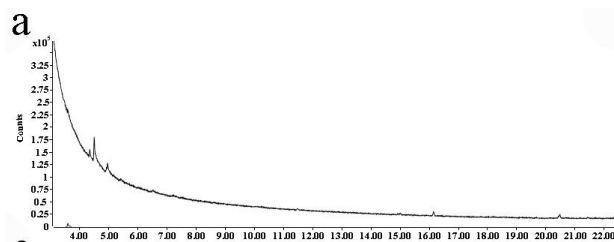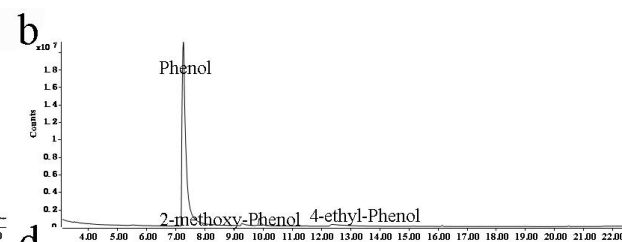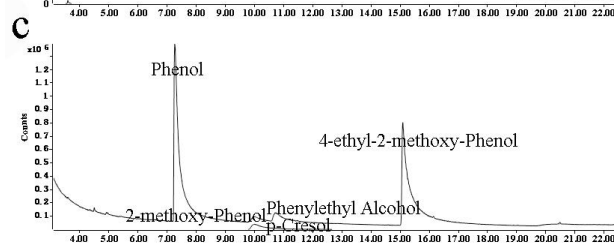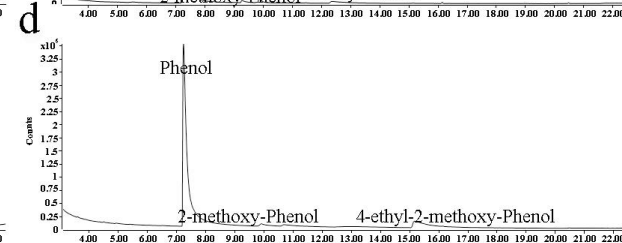

Supplement: Supplementary file 1 — Additional file 1: Figure S1. TIC of dichloromethane extract analysed as TMS derivative from control (A) and treatment CCJ (B), XCJ (C) and YCJ (D) in vitro. CCJ: gut symbiotic microbiota of female beetle; XCJ: gut symbiotic microbiota of male beetle; YCJ: gut symbiotic microbiota of beetle larvae. [file 13068_2019_1411_MOESM1_ESM.pdf]

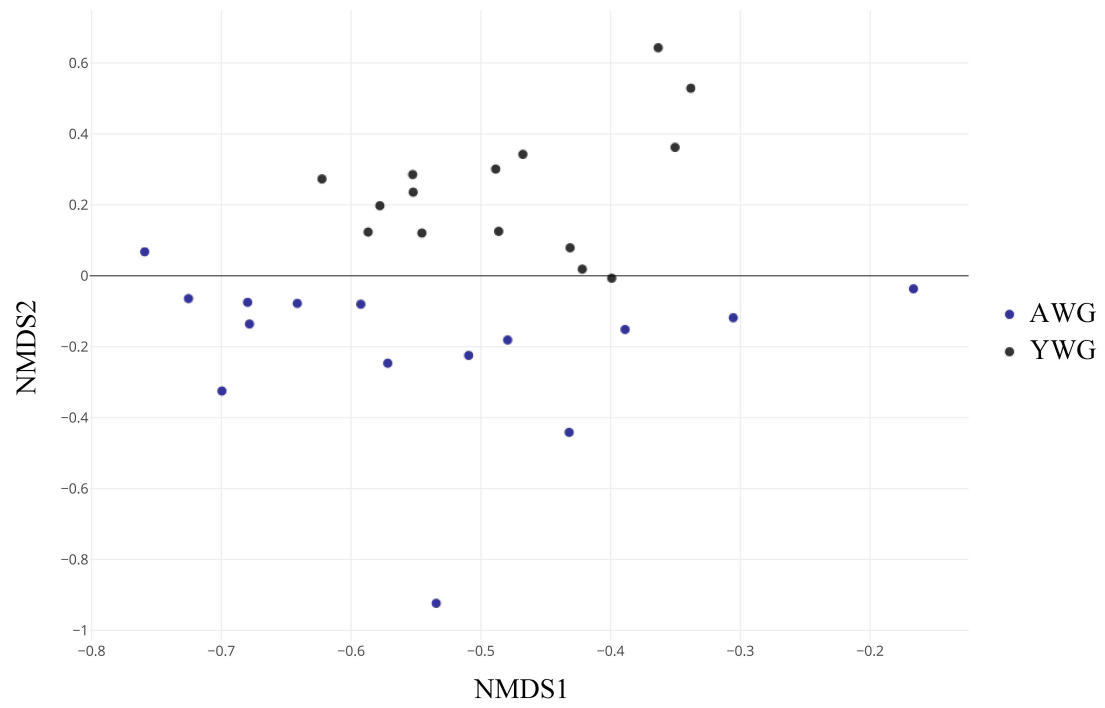

Supplement: Supplementary file 4 — Additional file 4: Figure S2. Non-metric multidimensional scaling (NMDS) analysis of the Bray–Curtis similarity coefficients based on the relative abundance of OTUs in the given sample. [file 13068_2019_1411_MOESM4_ESM.pdf]

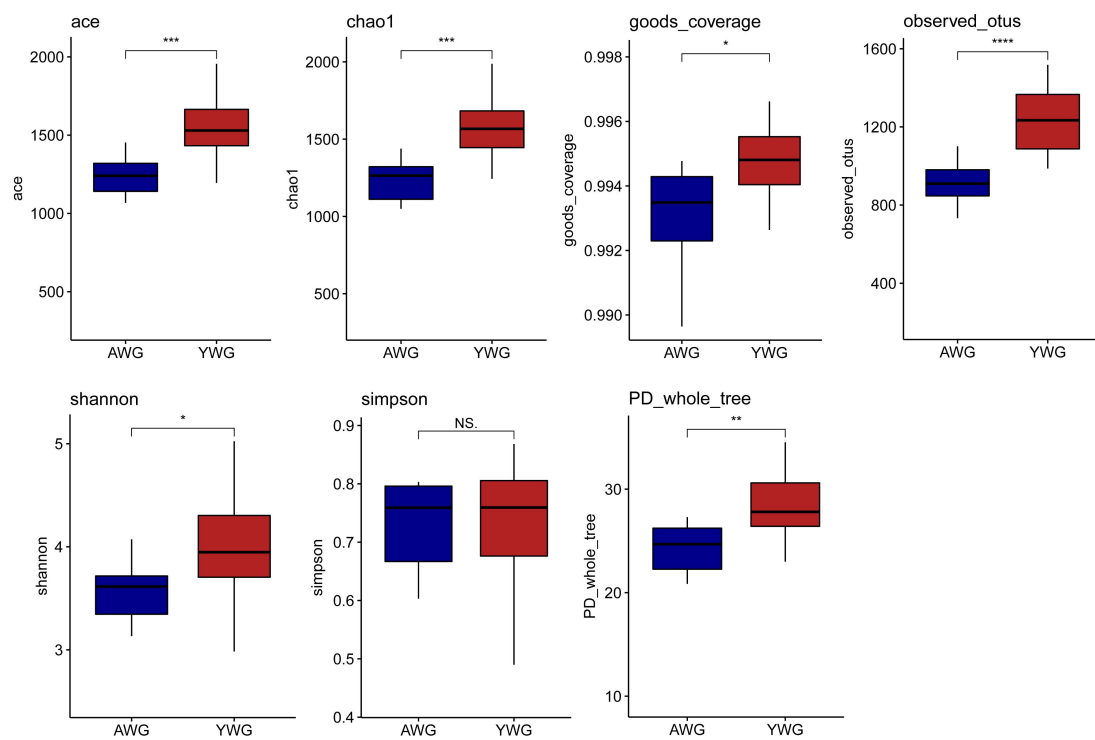

Supplement: Supplementary file 5 — Additional file 5: Figure S3. Boxplot analysis comparing the bacterial OTUs between the two groups. [file 13068_2019_1411_MOESM5_ESM.pdf]

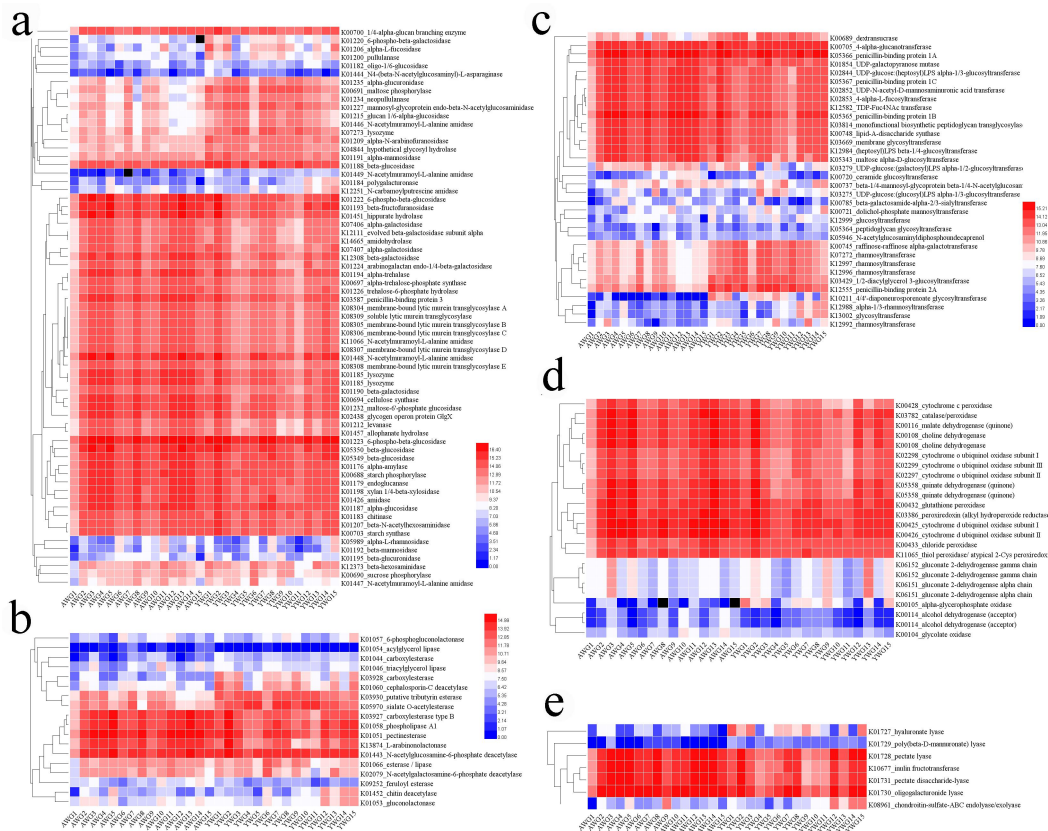

Supplement: Supplementary file 6 — Additional file 6: Figure S4. Relative abundance of PICRUSt-predicted CAZyme genes relevant to lignocellulose degradation. (A) Glycoside hydrolases (GHs). (B) Glycosyl transferases (GTs). (C) Carbohydrate esterases (CEs). (D) Carbohydrate-binding modules (CBMs). (E) Auxiliary activities (AAs). [file 13068_2019_1411_MOESM6_ESM.pdf]

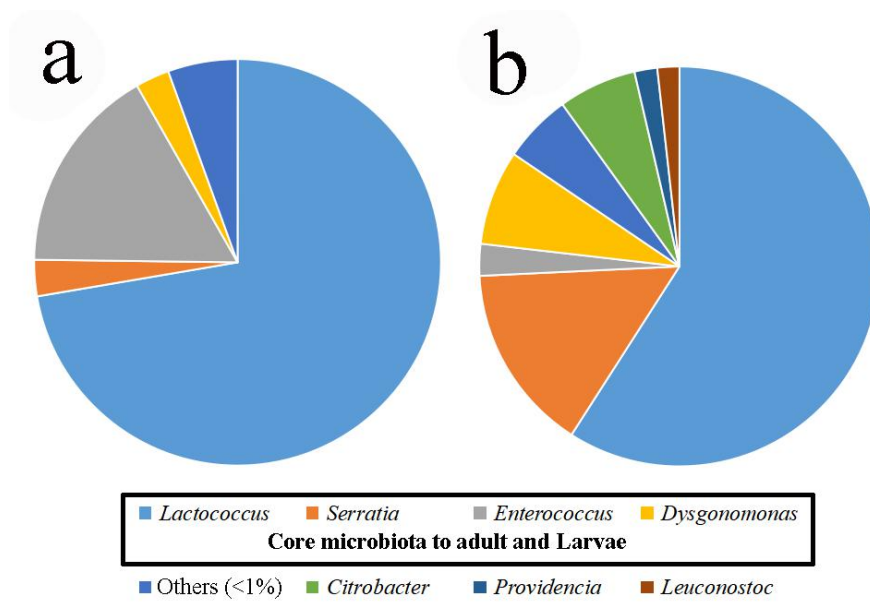

Supplement: Supplementary file 7 — Additional file 7: Figure S5. Composition of the digestive core microbiota at the genus level. [file 13068_2019_1411_MOESM7_ESM.pdf]
